# Formaldehyde Gas Sensors: A Review

**DOI:** 10.3390/s130404468

**Published:** 2013-04-02

**Authors:** Po-Ren Chung, Chun-Ta Tzeng, Ming-Tsun Ke, Chia-Yen Lee

**Affiliations:** 1 Department of Architecture, National Cheng Kung University, Tainan 701, Taiwan; E-Mails: benjamin@archilife.ncku.edu.tw (P.-R.C.); ctmt@mail.ncku.edu.tw (C.-T.T.); 2 Department of Energy and Refrigerating Air-conditioning Engineering, National Taipei University of Technology, Taipei 106, Taiwan; 3 Department of Vehicle Engineering, National Pingtung University of Science and Technology, Pingtung 912, Taiwan

**Keywords:** formaldehyde, gas sensor, indoor air, MEMS, sick building syndrome (SBS)

## Abstract

Many methods based on spectrophotometric, fluorometric, piezoresistive, amperometric or conductive measurements have been proposed for detecting the concentration of formaldehyde in air. However, conventional formaldehyde measurement systems are bulky and expensive and require the services of highly-trained operators. Accordingly, the emergence of sophisticated technologies in recent years has prompted the development of many microscale gaseous formaldehyde detection systems. Besides their compact size, such devices have many other advantages over their macroscale counterparts, including a real-time response, a more straightforward operation, lower power consumption, and the potential for low-cost batch production. This paper commences by providing a high level overview of the formaldehyde gas sensing field and then describes some of the more significant real-time sensors presented in the literature over the past 10 years or so.

## 1. Origin and Measurement Technology of Formaldehyde in Air

Formaldehyde is one of the volatile organic compounds (VOCs) that are widely used in household materials, which is associated with many health risk factors and has been identified as a major cause of sick building syndrome (SBS) [1–3]. SBS sufferers exhibit a range of symptoms which appear to be related to the time spent in a particular building [4]. The specific origins of SBS are not fully understood, but is thought that chemical and biological contaminants, and inadequate ventilation, all play a contributory role. Many of the upholstery, carpeting, wood and plastic products used in indoor environments emit VOCs, such as xylene and formaldehyde [5–13]. Consequently, the World Health Organization (WHO) has set a 30 min exposure limit of 0.08 ppm [14], while the US National Institute for Occupational Safety and Health (NIOSH) has established a maximum long-term exposure limit of 0.016 ppm (TWA) [15].

In the last two decades of the 20th century, a number of analytical methods for the determination of formaldehyde have been reported. These methods include spectrophotometry [16], gas chromatography (GC) [17], high-performance liquid chromatography [18], ion chromatography [19] and polarography [20]. Since these methods required expensive and bulky instrumentation with high power demand and well-trained operators, clearly, these procedures is unable to provide formaldehyde exposure information on a real-time basis.

To simplify the measurement of ambient formaldehyde, Hopkins *et al.* [21] proposed a GC-pulsed Helium Ionization Detector (pHID) apparatus aiming at formaldehyde detection and designed to operate at relatively high frequencies (>10 h^−1^). In order to maintain the simplicity of the apparatus, a back-flush system was used to prevent the build-up of water in the column and to increase the sample rate. However, the associated arrangements tend to be rather bulky and elaborate. With good selectivity and selectivity, miniaturized electrochemical sensors became available for detection of many different toxic gases in mid-1980s and were commercialized very soon. The sensors operate by reacting with the gas of interest and producing an electrical signal proportional to the gas concentration. However, the lifetime of an electrochemical sensor is highly dependent on the total amount of gas the sensor is exposed to during its lifetime, as well as other environmental conditions [22]. Over the past decade, emerging measurement technologies have contributed significantly to the miniaturization of measurement apparatus. As a result of advances in measurement technology, sensing instrumentation capable of accessing information at a real-time level is now available [10,22–48]. Reviewing the literature, it is found that the sensing mechanism of the majority of these sensors can be classified in as receptor-based or transducer-based formaldehyde sensors. The remainder of this paper presents a systematic review of the operational principles and advantages of each of the major formaldehyde gas sensors presented in the literature over the past 10 years or so.

**Figure 1.**
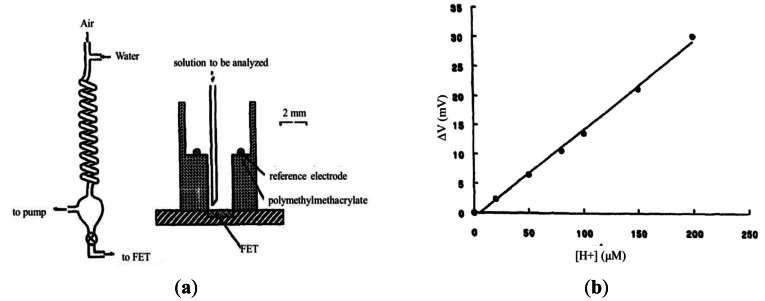
(**a**) Schematic illustration of FET-based detection of gaseous formaldehyde. **Left**: general arrangement of sampling system. **Right**: Detailed view of FET sensor. (**b**) Potentiometric response of FET sensor given increasing formaldehyde concentration [23].

In 1996, Vianello *et al.* [23] proposed a potentiometric formaldehyde detection system based on an aldehyde dehydrogenase ion-selective field effect transistor (ISFET, see Figure 1(a)). In the proposed approach, the atmospheric formaldehyde was dissolved in an aqueous solution and then deposited on the surface of an ISFET coated with the enzyme nicotinamide adenine dinucleoside (NAD). The formaldehyde concentration was then determined by measuring the change in the ISFET output signal during the subsequent reaction between the formaldehyde molecules and the enzyme. The experimental results showed that the sensor output voltage increased linearly with an increasing formaldehyde concentration (see Figure 1(b)). Moreover, it was shown that the minimum formaldehyde detection limit was around 0.1 ppm and stability was long, up to several months.

**Figure 2.**
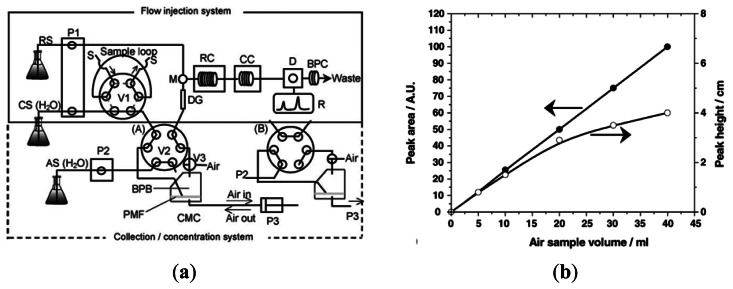
(**a**) Schematic illustration of flow injection system coupled with collection/concentration system for formaldehyde determination. (RS, reagent solution; CS and AS, carrier and absorbing solutions, respectively; P1, double-plunger pump; P2, peristaltic pump; P3, syringe pump; V1 and V2, six-way valves; V3, three-way valve; S, sample; M, mixing joint; DG, degassing unit; RC, reaction coil; D, detector; BPC, back-pressure coil; CMC, chromatomembrane cell; BPB, biporous PTFE block; PMF, porous membrane filter. (A) Introduction of absorbing solution into FIA system; (B) air sampling); (**b**) Variation of peak area and peak height with air sample volume [24].

Sritharathikhun *et al.* [24] presented a method for determining trace amounts of formaldehyde in air by coupling a three-hole chromatomembrane cell (CMC) and a flow injection analysis (FIA) system. As shown in Figure 2(a), the CMC was used to collect and concentrate trace amounts of gaseous formaldehyde in water and the resulting solution was then introduced into the carrier stream of the FIA system.

**Figure 3.**
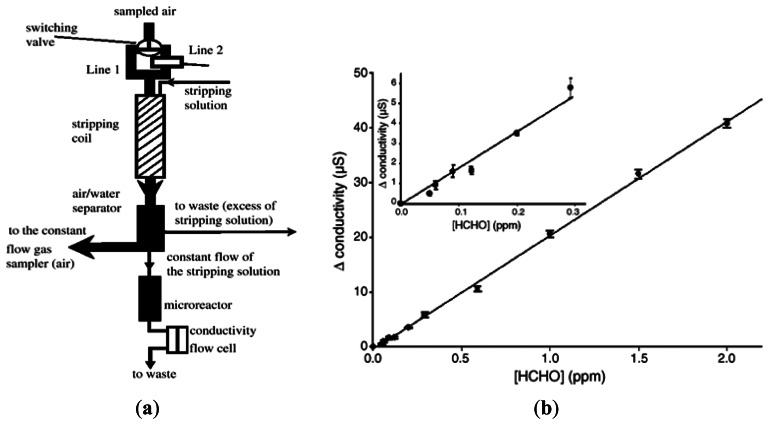
(**a**) Schematic illustration of biosensor setup. Atmospheric air is sampled by constant flow gas sampler and formaldehyde concentration is measured by sending air sample directly to scrubbing coil via switching valve (line 1). Note that blank signals are obtained by switching sampled air through HCHO trap containing 2,4-dinitrophenylhydrazine-loaded filter (line2). (**b**) Variation of conductivity with formaldehyde concentration [25].

Finally, the formaldehyde concentration was measured both spectrophotometrically and fluorometrically following a reaction between the solution and a mixed reagent of acetylacetone and ammonium acetate. The results obtained using an air flow rate of 6 mL min^−1^ and a sample size of 20 mL showed that the formaldehyde concentration in indoor air was equal to around 5.14 ppb (see Figure 2(b)). Vianello *et al.* [25] developed a system for the on-line detection of atmospheric formaldehyde comprising a wet scrubber, a micro-reactor containing immobilized formaldehyde dehydrogenase (FDH), and a conductometric transducer (see Figure 3(a)). In the proposed system, atmosphere was sampled by a constant flow gas sampler. Formaldehyde was measured sending the sampled atmosphere directly to the scrubbing coil by a switching valve (line 1). Blank signals were obtained by switching the sampled air through a HCHO trap containing 2,4-dinitrophenlhydrazine loaded filters (line 2). The HCHO stripping device was a glass coil. A 2-way peristaltic pump was used to control the solution flow rate. As shown in Figure 3(b), the system was capable of detecting formaldehyde concentrations of 0.05–2 ppm with a sensitivity of 20 μS/ppm. Though some devices were miniaturized quite a bit (e.g., the microreactor), the supporting system was still bulky and required to be reduced by some engineering tools.

In general, the gaseous formaldehyde detection systems presented in [23–25] have a high sensitivity and a wide measurement range, but they are complex. As a result, their use is limited to a laboratory environment. In practice, however, a requirement exists for low-cost, portable real-time measurement systems such that the formaldehyde concentration can be measured *in situ* (*i.e.*, without the need to collect samples and then transport them to a remote laboratory for testing purposes.) Accordingly, various small-scale formaldehyde measurement systems based on the use of sensing materials as receptors or transducers (Table 1) have been proposed in recent years [26].

## 2. Receptor-Based Formaldehyde Sensors

In general, receptor functional sensors transform chemical information into some form of energy which may be measured by a transducer [25]. As discussed in the following, existing proposals for gaseous formaldehyde detection using sensing materials as receptors can be broadly categorized as either spectrometric, piezoresistive or colorimetric, respectively.

### 2.1. Spectrometric Type

**Figure 4.**
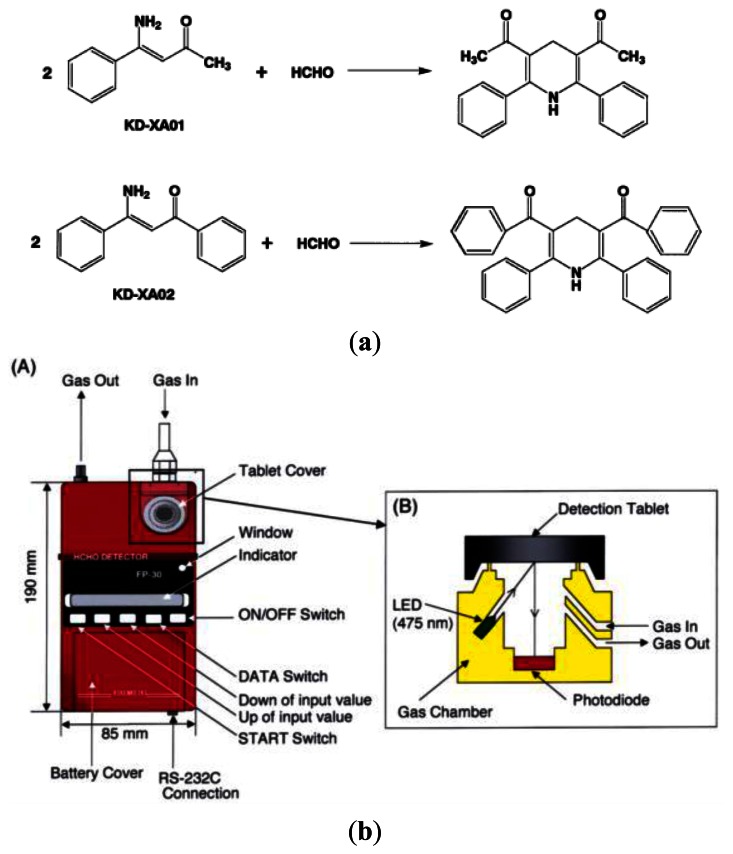
(**a**) Chemical structures of colorimetric reagents (KD-XA01 and KD-XA02) and their transformation into lutidine derivatives after reaction with formaldehyde. (**b**) (A) Schematic representation of the formaldehyde monitoring instrument and (B) the optical location of the LED and photodiode to detect the reflected light from the table in [27].

In the spectrometric type of formaldehyde sensors, sensing molecules produce speedy color changes from colorless to colored under mild conditions, which was caused by the fact that an enaminone structure in the reagent reacted with formaldehyde to give a lutidine derivative. In 2003, Suzuki *et al.* [27] developed a portable sick house syndrome gas monitoring system based on colorimetric reagents for the highly selective and sensitive detection of formaldehyde. In general, spectrometric formaldehyde gas sensors utilize colorimetric formaldehyde-sensing molecules which possessed an enaminone structure (Figure 4(a)).

**Figure 5.**
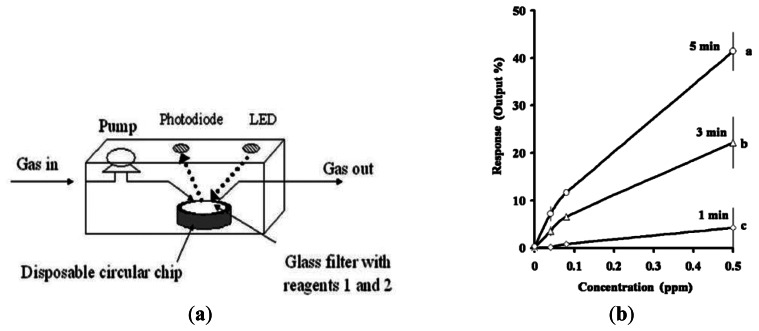
(**a**) Schematic illustration of formaldehyde sensor based on photometer and reagent-filled filter. (**b**) Variation of sensor response with formaldehyde concentration given sampling times of 1, 3 and 5 min [5].

The instrument (Figure 4(b)) detected the surface color change of the detection tablet from white to yellow, which was monitored as a function of the intensity of the reflected light illuminated by an LED (475 nm). The response was proportional to the formaldehyde concentration. Kawamura *et al.* [5] proposed a hand-held formaldehyde gas sensor comprising an LED light source (wavelength 540 nm) and a circular filter impregnated with potassium hydroxide solution and 100 μL of 4-amino hydrazine-5-mercapto-1,2,4-triazole (AHMT, see Figure 5(a)). When the filter was exposed to formaldehyde gas, the AHMT reagent reacted with the HCHO and resulted in a change in the color of the filter. The color change was then recorded by measuring the intensity of the light reflected from the surface of the filter using a photodiode. The results showed that a minimum detection limit of 0.04 ppm HCHO was possible given a sampling time of 3 min or more (see Figure 5(b)).

**Figure 6.**
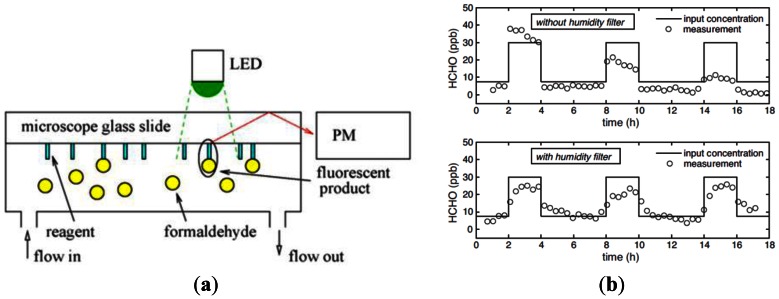
(**a**) Schematic illustration of formaldehyde sensor in which formaldehyde molecules react with Fluoral-P molecules to form DDL, which is then excited by LED with wavelength of 405 nm; (**b**) Pulse-mode detection of HCHO in atmosphere with relative humidity of 50% with and without humidity filter, respectively [28].

Descamps *et al.* [28] proposed a colorimetric device for measuring the concentration of gaseous formaldehyde incorporating a nanoporous film doped with Fluoral-P. When exposed to gaseous formaldehyde, the HCHO molecules reacted with the Fluoral-P reagent to form 3,5-diacetyl-1,4-dihydrolutidine (DDL). The formaldehyde concentration was then determined by measuring the intensity of the fluorescence emission signal given the use of a LED illumination light source with a wavelength of 405 nm (see Figure 6(a)). In computing the formaldehyde concentration, the DDL concentration was formulated as:

(1) [DDL]=a(1−exp(−bt))

where *a* and *b* include the reaction rate *k*, the initial concentration of Fluoral-P [*F*]_0_, and the formaldehyde concentration [HCHO], *i.e.*,:

(2) $$\begin{array}{c} a\propto {\left[F\right]}_{0}\\ b=k[\text{HCHO}] \end{array}$$

[F]_0_ was assumed to be constant throughout the experiments, and thus a X b was proportional to the formaldehyde concentration (Equation (2)). The experimental results showed a relative scattering of +20% for formaldehyde concentrations lower than 90 ppb and a minimum detection limit of 30 ppb (Figure 6(b)).

### 2.2. Piezoresistive Type

**Figure 7.**
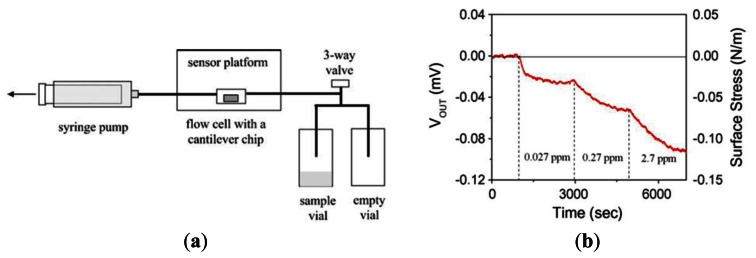
(**a**) Schematic illustration of formaldehyde sensor comprising piezoresistive cantilever sensor platform. (**b**) Variation of output voltage and surface stress over time given increasing concentration of formaldehyde vapor [29].

Piezoresistivity is a common sensing principle for micromachined sensors. Among all known piezoresistive materials, doped silicon, in particular, exhibits remarkable piezoresistive response characteristics. An electrical resistor may change its resistance when it experiences a strain and deformation. This effect provides an easy and direct energy/signal transduction mechanism between the mechanical and the electrical domains [22]. Seo *et al.* [29] proposed a gaseous formaldehyde sensor comprising a cantilever coated with a 3-mercaptophenol self-assembled monolayer (SAM, see Figure 7(a)).

It was shown that when the cantilever was exposed to interferents such as benzene, toluene, p-xylene, water or ethanol, the cantilever deflection was in the opposite direction to that when the cantilever was exposed to formaldehyde. Thus, the selectivity of the sensor was confirmed. Furthermore, as shown in Figure 7(b), the minimum detection limit of the sensor was determined to be 0.027 ppm.

### 2.3. Colorimetric Type

**Figure 8.**
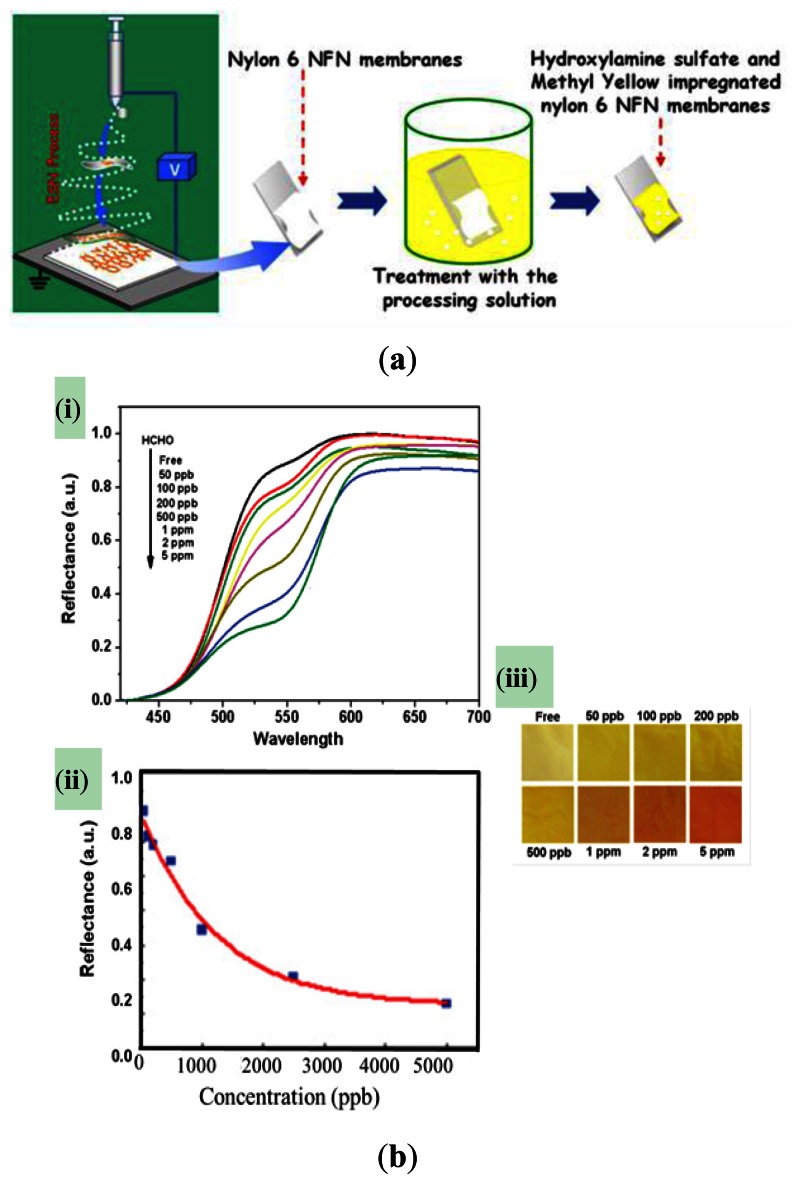
(**a**) Schematic illustration showing preparation of methyl yellow-impregnated Nylon 6 colorimetric NFN membranes. (**b)** (i) and (ii) Variation of reflectance with wavelength as function of formaldehyde concentration, and (iii) color-differentiation map comprising converted RGB colors for various formaldehyde concentrations [13].

The colorimetric type of sensors presents a decrease of reflectance intensity at a specific excitation wavelength. The color of the illuminated filter changes as the formaldehyde concentration changes. Wang *et al.* [13] proposed a colorimetric sensor for the detection of formaldehyde based on methyl yellow-impregnated electro-spinning/netting (ESN) Nylon 6 nano-fiber/nets (NFN, see Figure 8(a)).

When exposed to gaseous formaldehyde, the following reaction occurred:

(3) $$2\text{HCHO}+{\left(NH_{2}OH\right)}_{2}•H_{2}{\text{SO}}_{4}\to 2H_{2}C=\text{NOH}+H_{2}{\text{SO}}_{4}+2H_{2}O$$

The sensor exhibited a significant decrease in the reflectance intensity at an excitation wavelength of 550 nm. Moreover, it was shown that the color of the illuminated filter changed from yellow to red as the formaldehyde concentration was increased from 50 ppb to 5 ppm (see Figure 8(b)).

**Figure 9.**
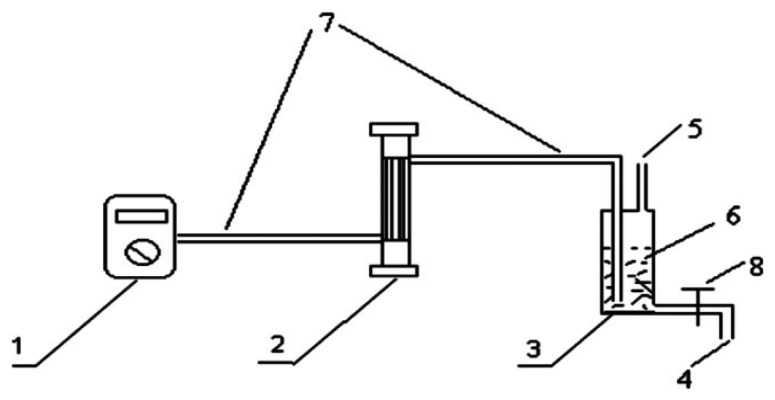
Gaseous formaldehyde detection system comprising pump (1), rotameter (2), reservoir bottle (3), solution exit (4), exit and solution entrance (5), ammonium sulfate solution (6), pipe (7) and piston with hole (8) [30].

Deng *et al.* [30] presented a formaldehyde gas sensor based on an ammonium sulfate derivatization reagent and a capillary electrophoresis – electrochemical detection (CEED) system (see Figure 9). The reaction between the derivatization regent and formaldehyde was formulated as follows:

(4) $$4NH_{4}++6\text{HCHO}↹{\left(CH_{2}\right)}_{6}N_{4}H^{+}+3H^{+}+6H_{2}O.$$

It was shown that the intensity of the detection signal varied linearly with the formaldehyde concentration over the range of 0.4 ppb to 770 ppb. Moreover, the minimum detection limit was shown to be 0.12 ppb.

In general, formaldehyde gas sensors based on sensing material reagents have two major advantages compared to their previous counterparts (e.g., detection in solution state), namely (1) a lower detection limit, and (2) a more rapid response. However, they have a short lifetime since the sensing material is gradually consumed during the reaction process.

## 3. Transducer-Based Formaldehyde Sensors

Recently, many formaldehyde gas sensors incorporating micro- or nano-fabricated sensing materials as transducers have been proposed. The transducer functional sensors transform energy carrying the chemical information about the sample into a useful analytical signal [26]. Broadly speaking, these sensors can be categorized as either amperometric or conductimetric.

### 3.1. Amperometric Type

**Figure 10.**
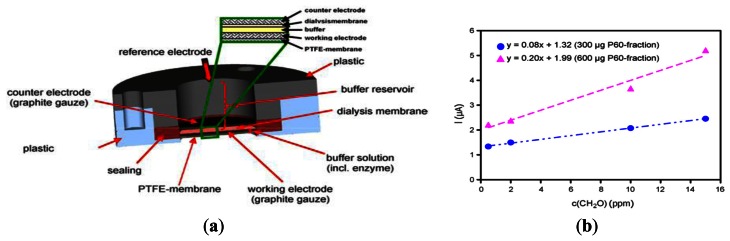
(**a**) Sensor for gaseous formaldehyde detection comprising multiple membranes and electrodes. (**b**) Characteristic response curve of sensor given different enzyme loads [31].

**Figure 11.**
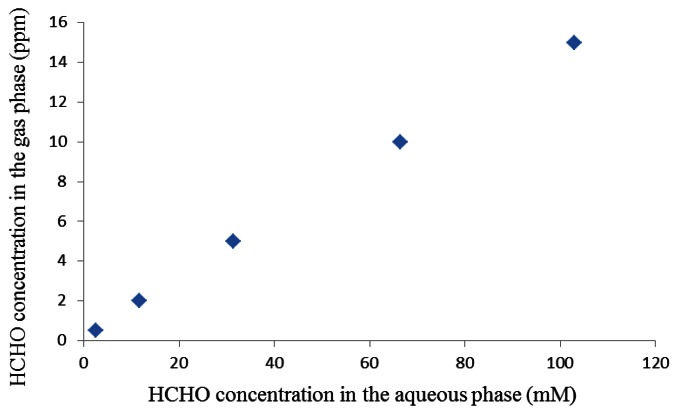
Formaldehyde concentration in the aqueous phase and the corresponding equilibrium gas phase concentrations at 20 °C according to the equation given in [32].

The detection of formaldehyde molecules presence in air on the basis of electric current or change in electric current is called an amperometric formaldehyde gas sensor. Achmann *et al.* [31] proposed an amperometric enzyme-based sensor-system for the detection of formaldehyde in air based on a native bacterial enzyme (NAD^+^-) and glutathione-independent formaldehyde dehydrogenase (see Figure 10(a)). The sensor device consisted of a 3-electrode configuration with disks of woven graphite gauze as working and counter electrode included in a plastic housing. Both electrodes were contacted with Pt-wire. The gas diffused into the liquid phase via a 15 mm diameter PTFE membrane. Gaseous formaldehyde samples were collected from the headspace above aqueous solutions of known concentration. The formaldehyde concentration in the gas phase above the solution was calculated according to the equation given by Dong *et al.* [32], as shown in Figure 11. It was shown that the sensitivity of the device was more than 90% and a 98.5% reproducibility of the sensor signal after 14 h at 4 °C. However, the minimal detection limit was just 0.5 ppm (see Figure 10(b)).

**Figure 12.**
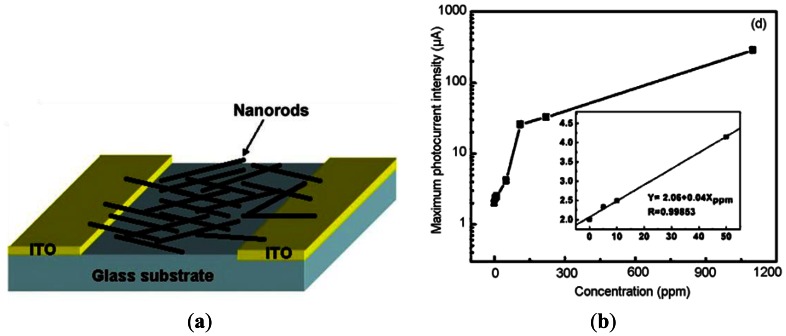
(**a**) Formaldehyde sensor comprising nano-rods deposited on ITO/glass substrate; (**b**) Variation of photocurrent intensity with formaldehyde concentration [33].

Peng *et al.* [33] developed a formaldehyde gas sensor comprising ZnO nanorods deposited on an ITO/glass substrate (see Figure 12(a)). The sensing characteristics of the proposed device were investigated both with and without ultraviolet (UV) irradiation. The UV-assisted formaldehyde sensing was demonstrated by detecting the photocurrent intensity change as the ZnO nanorods were exposed to formaldehyde. The enhancement of formaldehyde-response corresponded to the photocatalytic oxidation which was caused by the oxygen adsorbed on nanorods surface. The experimental results showed that the response of the nanorods to 110 ppm of formaldehyde was around 120 times higher with UV light irradiation than without UV light irradiation. However, the minimal detection limit was just 1.8 ppm (see Figure 12(b)).

### 3.2. Conductimetric Type

The transduction mechanism of conductimetric formaldehyde gas sensors involves the changes in conductivity caused by the adsorption of formaldehyde gas. In the last decade, the detection of formaldehyde has been developed to provide formaldehyde exposure information on a real-time basis. Metal-oxide semiconductors (MOXs) are cheap and common catalysts used to induce the oxidation formaldehyde.

**Figure 13.**
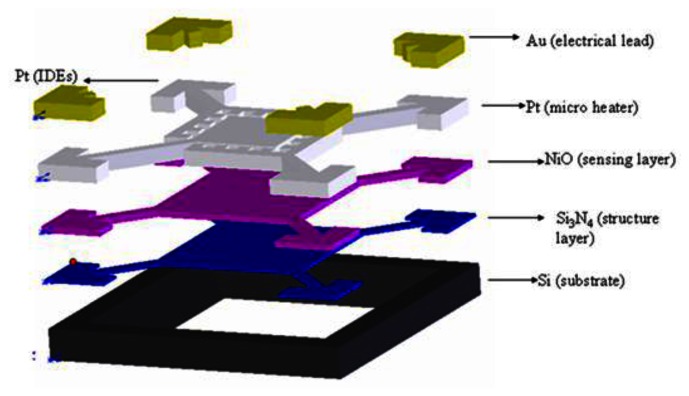
Schematic illustration of formaldehyde sensor comprising integrated micro-hotplate and IDEs [41].

In MOX sensors, electron donors or acceptors in the gas phase adsorb onto the metal oxide. At high temperature (>200 °C), the adsorbed species can exchange electrons with the metal oxide. An electron donor increases the conductivity, while an acceptor molecule takes electrons and reduces its conductivity [33]. Commonly used materials include ZnO [34–38], WO_3_ [39], hybrid materials [40], NiO [41–44] and so on. Lee *et al.* [41] presented a gaseous formaldehyde sensor comprising a suspended silicon nitride microstructure with an integrated micro Pt heater, a thin-film NiO sensing layer, and Pt interdigitated electrodes (IDEs) (see Figure 13).

It was shown that in the presence of gaseous formaldehyde, an oxidation process occurred near the heated NiO sensing layer, which caused a change in the electrical conductivity of the NiO film and therefore changed the resistance between the interdigitated electrodes. It was further shown that the change in resistance varied linearly with the formaldehyde concentration in the range of 0–5 ppm. Thus, by measuring this change in resistance, the formaldehyde concentration could be inversely derived.

**Figure 14.**
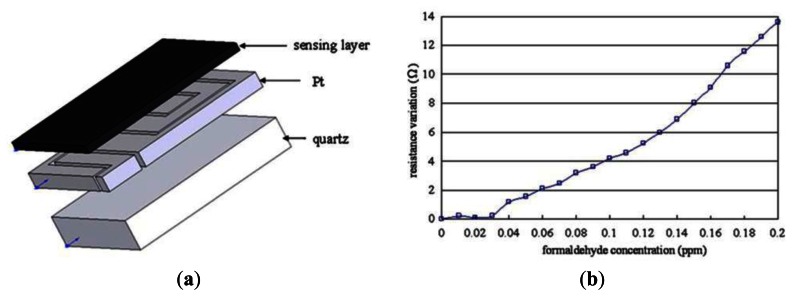
(**a**) Schematic illustration of formaldehyde sensor comprising sensing layer deposited on micro-hotplate. (**b**) Variation of resistance with formaldehyde concentration [44].

Dirksen *et al.* [42] examined all of the catalysts used for the oxidation of formaldehyde, and reported that the most active catalytic oxide appeared to be NiO. It was shown that the electrical conductivity of NiO depended significantly on the partial pressure of oxygen in the atmosphere. Moreover, it was shown that this phenomenon could be exploited to sense the concentration of gaseous formaldehyde by measuring the change in the electrical conductivity of the NiO oxide during the catalytic oxidation process. Wang *et al.* [44] proposed a MEMS-based formaldehyde gas sensor consisting of a thin-film NiO/Al_2_O_3_ sensing layer deposited on a Pt-based hotplate (see Figure 14(a)).

The experimental results showed that both the sensitivity of the sensor and the minimal detection limit could be improved by increasing the area of the sensing surface or reducing the thickness of the sensing layer. As shown in Figure 14(b), the minimal detection limit was found to be 40 ppb given the use of a hybrid NiO/Al_2_O_3_ sensing layer.

**Figure 15.**
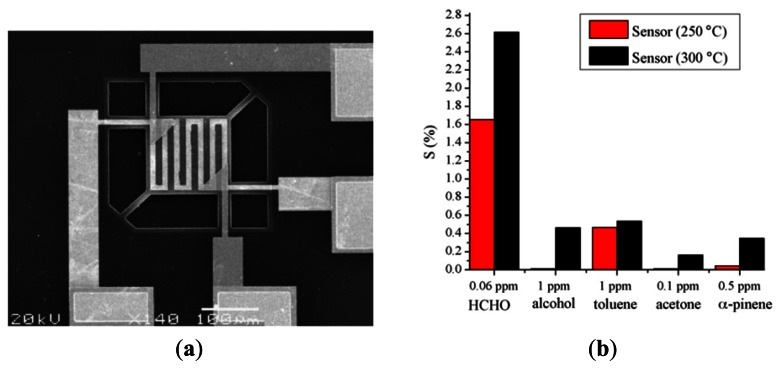
(**a**) SEM image of micro-hotplate within dual-sensor detection chip. (**b**) Output response of sensor in presence of various compounds with different concentrations [45].

Lv *et al.* [45] developed a formaldehyde gas sensor incorporating a thin film of SnO_2_-NiO nanometer polycrystalline composite deposited on a micro-hotplate (MHP) (see Figure 15(a)). It was shown that the device was capable of detecting gaseous concentrations as low as 0.06 ppm given a MHP working temperature of 300 °C. Moreover, the device showed good selectivity in the presence of common interferents such as alcohol, toluene, α-pinene and acetone (see Figure 15(b)). Gastro-Hurtado *et al.* [46] presented a NiO thin film formaldehyde gas sensor similar to that proposed by Lee *et al.*[41] and Wang *et al.* [44]. The same group also developed a gaseous formaldehyde sensor based on SnO_2_-nanowires grown by the catalytic oxidation of Sn-sputtered thin films [47]. The experimental results presented in Ref [47] showed that the addition of metal catalyst materials such as Au and Pt improved the sensor response and reduced the device operating temperature to 130 °C.

Recently, carbon nanotubes (CNTs) have been widely used as sensing materials to detect low concentrations of gases, such as nitrogen oxides, ammonia, hydrogen, carbon monoxide and some organic gases, due to their specific properties of nanometer hollow geometry, high specific surface area, high electron mobility, surface modification and functionalization [48]. Though a low concentration of formaldehyde (20 ppb) could be attained, the selectivity is still concerned in the development of gas sensors based on CNTs.

## 4. Conclusions

Technical advances in recent years now make possible the fabrication of sophisticated sensors for a diverse range of applications. Compared to their traditional macroscale counterparts, microscale sensors generally have greater sensitivity, lower cost, improved portability and more straightforward integration with IC circuit devices. This paper has presented a systematic review of the most significant formaldehyde gas sensors presented in the literature over the past 10 years or so. It has been shown that these sensors can be broadly categorized as “receptor-” or “transducer-” based formaldehyde sensors, respectively. The operational principles and sensing performance of each type of sensor have been discussed, and their relative advantages and disadvantages described where appropriate. Overall, the results presented in this review confirm the applicability of recently developed and advanced sensors for a diverse range of low-cost, high-performance gas sensing applications.

## Figures and Tables

**Table 1.** Performance of small-scale formaldehyde gas sensors.

**Year**	**Authors**	**Functional Principle**	**Sensing Materials Working Temperature**	**Sensitivity Measurement Range**	**Reference Number**
2003	Suzuki *et al.*	Receptor	Colorimetric reagents	0.13 a.u./ppm	[27]
			35 °C	0–1.0 ppm	
2007	Seo *et al.*	Receptor	Mercaptophenol	0.37 mV/ppm	[29]
			No data	0.027–2.7 ppm	
2007	Lee *et al.*	Transducer	NiO	0.33 Ω/ppm	[41]
			300 °C	0–30 ppm	
2008	Achmann *et al.*	Transducer	Enzyme	390 nA/ppm	[31]
			No data	0.5–15 ppm	
2008	Lv *et al.*	Transducer	SnO_2_-NiO	0.53 ppm^−1^ (R_a_/R_g_)	[45]
			300 °C	0.06–0.3 ppm	
2008	Bai *et al.*	Transducer	ZnO	10.6 a.u./100ppm	[34]
			420 °C	0–100 ppm	
2008	Wang *et al.*	Transducer	NiO-Al_2_O_3_	70 Ω/ppm	[44]
			300 °C	0–15 ppm	
2009	Chu *et al.*	Transducer	ZnO	2.11 ppm^−1^ (R_a_/R_g_)	[35]
			210 °C	1–10 ppm	
2009	Peng *et al.*	Transducer	ZnO	0.04 μA/ppm	[33]
			25 °C	0–50 ppm	
2010	Xie C *et al.*	Transducer	ZnO_-_MnO_2_	1.02 a.u./ppm	[40]
			320 °C	10–300 ppm	
2011	Han *et al.*	Transducer	ZnO	10 a.u./ppm	[36]
			200 °C	0–200 ppm	
2011	Castro-Hurtado *et al.*	Transducer	NiO	2.53 × 10^3^Ω/ppm	[46]
			340 °C	5–20 ppm	
2011	Zhang *et al.*	Transducer	ZnO	0.564 ppm^−1^ (R_a_/R_g_)	[37]
			400 °C	1–1000 ppm	
2011	Descamps *et al.*	Transducer	Fluoal-P	1.2 × 10^−5^ Vs^−1^/ppb	[28]
			Room temperature	0–200 ppb	
2012	Castro-Hurtado *et al.*	Transducer	SnO_2_	10 MΩ/ppm	[47]
			130 °C	0.5–15 ppm	
2012	Deng L *et al.*	Transducer	WO_3_	3.7 × 10^−10^ (Ωs)^−1^/ppm	[39]
			Room temperature	10–100 ppm	
2012	Deng B *et al.*	Receptor	(NH_3_)_2_SO_4_	No data	[30]
			No data	0.48–96,000 mg/m^3^	
2012	Xie H at al.	Transducer	Carbon nanotube	0.4 ppm^−1^ (R_a_/R_g_)	[48]
			Room temperature	0–50 ppb	
